# Novel Transparent Films Composed of Bisphenol-A Polycarbonate and Copolyester

**DOI:** 10.3390/polym14194146

**Published:** 2022-10-03

**Authors:** Hiroyuki Hasegawa, Takumitsu Kida, Masayuki Yamaguchi

**Affiliations:** 1 School of Materials Science, Japan Advanced Institute of Science and Technology, Asahidai, Nomi 923-1292, Japan; 2 Research & Business Development Center, Dai Nippon Printing Co., Ltd., Midorigahara, Tsukuba 300-2646, Japan

**Keywords:** polymer blend, transparent film, polycarbonate, polyester

## Abstract

In this paper, the structure and properties of transparent films composed of bisphenol-A polycarbonate (PC) and a commercially available copolyester, poly(1,4-cyclohexanedimethanol-*co*-2,2,4,4-tetramethyl-1,3-cyclobutanediol-*co*-terephthalate) (CPE), were studied. Both PC and CPE films are known to be transparent with good mechanical toughness. It was found that PC/CPE (50/50) showed miscibility in both the molten and solid states, indicating that there is a high possibility for the blend system to be miscible in the whole blend ratios. Because of the miscibility, the blend films showed no light scattering originating from phase separation. The mechanical properties of the films, such as Young’s modulus, yield stress, and strain at break, were determined by the blend ratio, and the glass transition temperature increased with the PC content, which corresponded well with the values predicted by the Fox equation. These results demonstrate that the thermal and mechanical properties of the films can only be controlled by the blend ratio. Since these transparent films showed excellent mechanical toughness irrespective of the blend ratios, they can be employed in various applications.

## 1. Introduction

Polycarbonate (PC) derived from bisphenol-A is one of the most successful transparent plastics because of its excellent impact resistance, transparency, and heat resistance [1,2,3]. However, PC has disadvantages in flowability due to its high glass transition temperature *T*_g_ and poor surface hardness. Therefore, PC is often mixed with another polymer species, such as acrylonitrile–butadiene–styrene (ABS) terpolymers [4,5,6,7], methyl methacrylate–butadiene–styrene (MBS) terpolymers [4,5], poly(lactic acid) [4,8,9,10], and polypropylene [4,11,12], in industry. These polymer blends always lose their transparency because of phase separation. It is known that poly(methyl methacrylate) (PMMA) with a low molecular weight can be miscible with PC and provides good flowability with enhanced scratch resistance [13,14,15,16], although the blends show phase separation at high temperatures, i.e., beyond lower critical solution temperatures (LCSTs) and under high pressure [17]. Once phase separation occurs, the blends become opaque due to the huge difference in the refractive index. Moreover, mechanical toughness, one of the most attractive properties of PC, decreases with the PMMA content, because low-molecular-weight PMMA dilutes the entanglement density of PC.

Blending with polyesters is another approach to enhancing flowability. PC is known to be immiscible with most conventional polyesters, including poly(ethylene terephthalate) (PET) [18], although Nassar et al. reported that PET-rich blends are miscible [19]. In many cases, however, blends of PC and polyester are opaque due to light scattering originating from the phase-separated structure. Therefore, in a previous study, a glycidyl compound, as a reactive compatibilizer, was mixed to improve transparency [20,21,22]. It is usually difficult to use such reactive blends in film applications because optically heterogeneous particles called “fish-eyes” often appear due to localized excess reactions. The transesterification reaction, which is enhanced by adding a tin compound as a catalyst [23,24,25], has also been employed to prepare a transparent blend film. Although the transesterification reaction can be inhibited by the addition of a phosphite compound, the molecular weight after the reaction is usually very low, leading to poor mechanical toughness.

Meanwhile, poly(1,4-cyclohexanedimethylene terephthalate) and poly(1,4-cyclohexanedimethylene-*co*-terephthalate-*co*-isophthalate) have been found to be miscible with PC, even without reactive mixing [26]. Moreover, blends of PC and poly(ethylene terephthalate-*co*-1,4-dimethyl cyclohexane terephthalate) have an upper critical solution temperature (UCST)-type phase diagram [27]. These reports should be noted because no chemical reaction is necessary in the system, which makes application in industry easier. Recently, a new type of copolyester named Tritan^TM^ has become commercially available from Eastman Chemical Company [28]. It shows good flowability and impact resistance. Furthermore, its heat resistance is relatively good, although PC shows better heat resistance. Since this copolyester contains a large amount of cyclohexanedimethylene, there is a possibility that it is miscible with PC.

Here, we investigated the miscibility of binary blends of PC and poly(1,4-cyclohexanedimethanol-*co*-2,2,4,4-tetramethyl-1,3-cyclobutanediol-*co*-terephthalate) at various blend ratios. Moreover, the transparency, viscoelastic properties, and mechanical properties of the films were examined. Recently, a large number of studies have been conducted in the field of functional polymer films [29,30,31,32,33]. We hope that the results of the present research will lead to significant findings in the future.

## 2. Materials and Methods

### 2.1. Materials

A commercially available PC (Iupilon S3000, Mitsubishi Engineering-Plastics Corp., Tokyo, Japan) was employed. The number- and weight-average molecular weights of PC, evaluated by size exclusion chromatography (Acquity APC System, Waters Corp., Milford, MA, USA) with polystyrene standards, were *M_n_* = 2.0 × 10^4^ and *M_w_* = 4.2 × 10^4^, respectively. Furthermore, a commercially available copolyester (CPE), poly(1,4-cyclohexanedimethanol-*co*-2,2,4,4-tetramethyl-1,3-cyclobutanediol-*co*-terephthalate), produced by the Eastman Chemical Company (Kingsport, TN, USA) as Tritan^TM^, was another polymer used in this study. The average molecular weights of CPE were evaluated by size exclusion chromatography (GPC-104, Shoko Science Co., Ltd., Yokohama, Japan) with PMMA standards, and they were found to be *M_n_* = 5.7 × 10^3^ and *M_w_* = 1.3 × 10^4^. The monomer composition in CPE, determined by ^13^C-nuclear magnetic resonance, was as follows: 52.8 mol% of terephthalic acid, 36.7 mol% of 1,4-cyclohexanedimethanol, and 10.5 mol% of 2,2,4,4-tetramethyl-1,3-cyclobutanediol.

### 2.2. Sample Preparation

After vacuum drying at 80 °C for 4 h, melt blending was performed using an internal mixer (HAAKE PolyLab OS, Thermo Fisher Scientific Inc., Waltham, MA, USA). The mixing was carried out at 260 °C for 3 min, with a blade rotation speed of 100 rpm. The weight fractions of CPE were 0, 10, 20, 50, 80, 90, and 100 wt%.

The obtained mixtures were compressed into flat films with various thicknesses using a compression molding machine (MH10, Imoto Machinery Co., Ltd., Kyoto, Japan) after vacuum drying at 80 °C for 4 h. The samples were melted for 4 min with slight pressure at 260 °C, followed by an applied pressure of 10 MPa for 30 s. Subsequently, the samples were quenched at 25 °C.

### 2.3. Measurements

The transparency of the films with 80 μm thickness was evaluated using a UV–vis spectrometer (UV-2700, Shimadzu Corp., Tokyo, Japan). The light transmittance was measured from 300 to 800 nm at 25 °C.

The wavelength dispersion of the refractive index was evaluated with an Abbe refractometer (DR-M2, Atago Co., Ltd., Tokyo, Japan) at 25 °C using compression-molded films. As a contact liquid, α-bromonaphthalene was employed.

Blend morphology was observed using a transmittance electron microscope (TEM; S-4800 Type II, Hitachi High-Tech Corp., Tokyo, Japan) at an accelerated voltage of 30 kV using an ultra-thin slice of 80 μm of PC/CPE (50/50) after staining with ruthenium tetroxide.

The temperature dependence of the dynamic tensile modulus in the solid state was measured using a dynamic mechanical analyzer (RSA-G2, TA instruments, New Castle, DE, USA). The applied frequency was 10 Hz, and the temperature range was from −150 °C to 200 °C, with a heating rate of 3 °C/min. Rectangular specimens with 600 μm thickness were cut out from the compression-molded films. The measurement was performed one time for each sample because of its good reproducibility.

The angular frequency dependence of the oscillatory shear modulus in the molten state was measured using a cone-and-plate rheometer (MCR301, Anton Paar Co., Ltd., Graz, Austria) under a nitrogen atmosphere at various temperatures.

Tensile tests were performed at 25 °C using a universal testing machine (Autograph AG-X, Shimadzu Corp., Tokyo, Japan). Dumbbell-shaped test pieces with 2 mm thickness were cut from the films obtained by compression molding. The initial distance between chucks was 60 mm. All tests were performed at a crosshead speed of 50 mm/min.

## 3. Results and Discussion

### 3.1. Transparency of the Films

**Figure 1:**
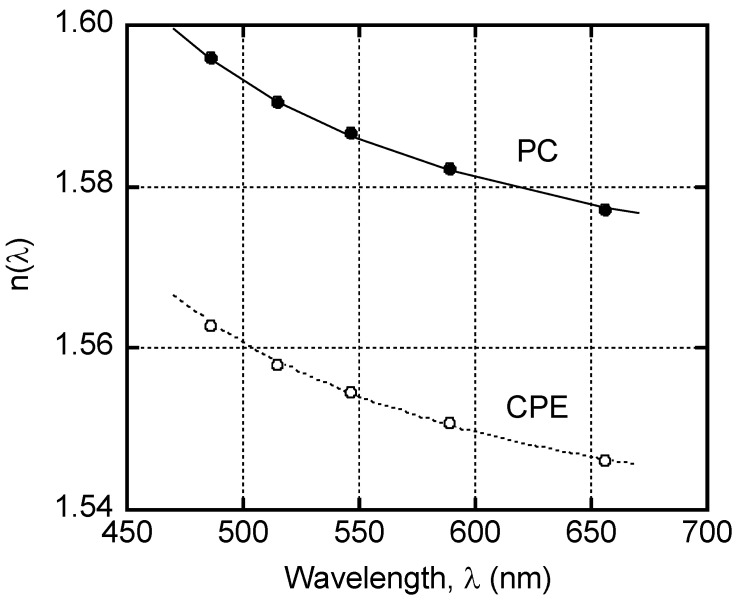
Wavelength dispersion of refractive index *n* (*λ*) for PC and CPE. The solid and dotted lines represent the calculated values using the Sellmeier equation.

Figure 1 shows the refractive indices *n* of the films of pure PC and CPE as a function of wavelength *λ*. PC showed a high refractive index as previously reported [34]. Moreover, CPE also showed high values, which is attributed to the terephthalate unit being used as acid. However, there remains a difference in the refractive index between PC and CPE in the wide range of wavelength. Because of the refractive index difference, the blend films must be opaque once phase separation occurs [35].

The wavelength dispersion of the refractive index is generally expressed by the Sellmeier equation [36],

(1) $$n\left(\lambda \right)=A+\frac{B}{\lambda^{2}-\lambda_{ab}{}^{2}}$$

where *λ_ab_* is the wavelength of a strong vibrational absorption peak in the ultraviolet region, and *A* and *B* are the Sellmeier coefficients. The lines in the figure denote the calculated values following Equation (1). The experimental data demonstrated that both PC and CPE exhibited a similar wavelength dispersion of the refractive index. This result indicates that the absorption peaks are located at a similar wavelength [37]. Based on the fitting curves using Equation (1), the peak was located at around 290 nm.

**Figure 2:**
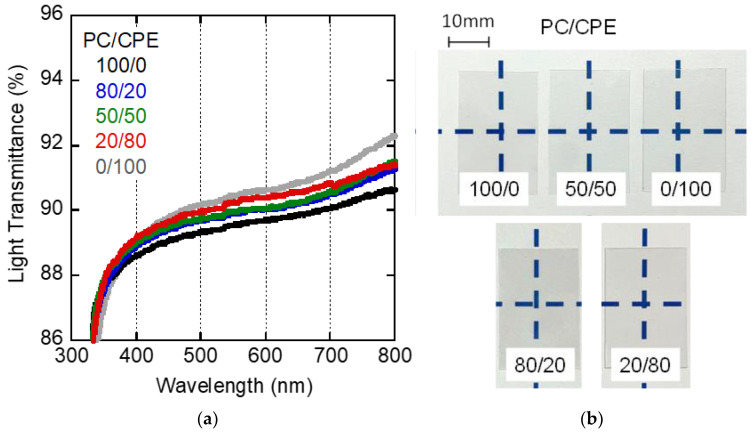
(**a**) Wavelength dispersion of light transmittance of the films with (**b**) optical photographs of PC, CPE, and their blend films.

The light transmittance of the films with 80 μm thickness is shown in Figure 2 with optical photographs. The light transmittances of pure PC and CPE were around 88% and 90%, respectively, at 589 nm. Since both pure polymers have almost no light scattering or absorption, the transmittance is mainly determined by the surface reflection given by the following equation [38]:

(2) $$R_{ref}={\left(\frac{n_{film}-n_{air}}{n_{film}+n_{air}}\right)}^{2}$$

where *R_ref_* is the reflectivity, and *n_film_* and *n_air_* are the refractive indices of the film and air (≈1), respectively. The slightly high values of the light transmittance of CPE must be attributed to the low reflectivity.

Furthermore, it should be noted that the blend films were transparent, which was confirmed from the photographs. The light transmittance was between that of the pure PC and CPE films. As previously mentioned, the refractive index difference between PC and CPE results in light scattering once phase separation occurs [35]. Therefore, these results suggest that the blends were in the miscible state.

It was also found from the figure that the light transmittance for all films greatly decreased with a decrease in the wavelength at around 350 nm. Since *λ_ab_* was almost the same for PC and CPE, this is a reasonable result.

### 3.2. Morphology Observation

**Figure 3:**
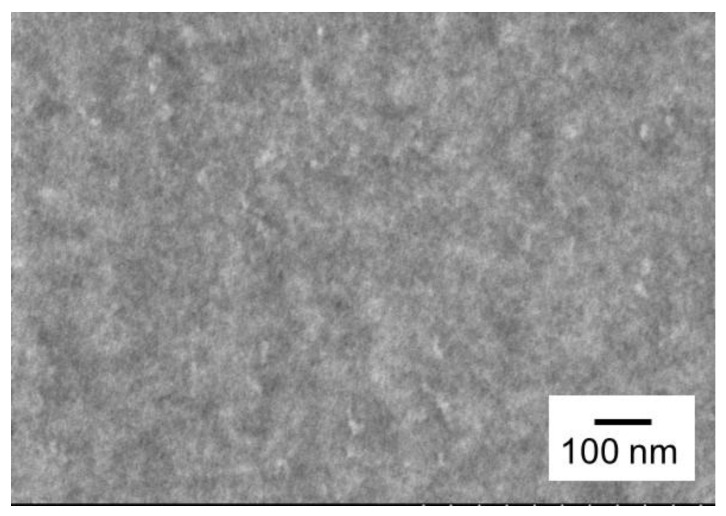
TEM image of PC/CPE (50/50).

A TEM image of PC/CPE (50/50) is shown in Figure 3. The sample was stained with ruthenium trioxide before observation. It was found that there was no phase-separated structure in the blend. Considering the uncertainty of an appropriate staining condition, however, we cannot conclude that the blend is miscible only from the image. Therefore, we further examined the miscibility by measuring the linear viscoelastic properties in both the solid and molten states.

### 3.3. Viscoelasticity in the Solid State

**Figure 4:**
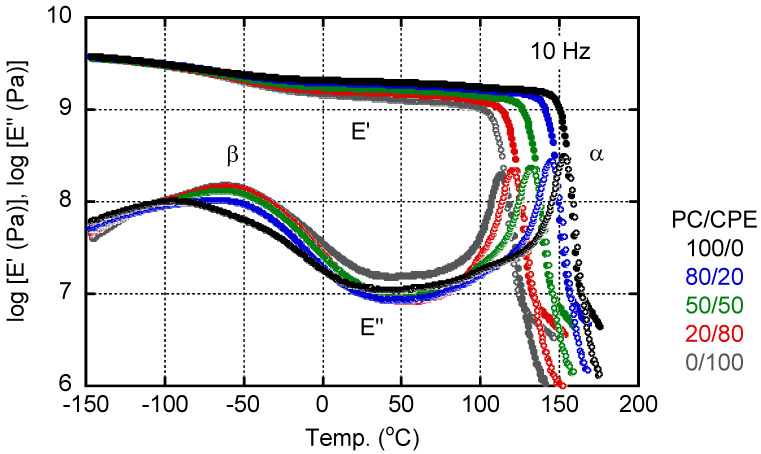
Temperature dependence of tensile storage moduli *E*′ and loss modulus *E*″ at 10 Hz.

Figure 4 shows the temperature dependence of the tensile storage modulus *E*′ and the loss modulus *E*″ at 10 Hz. All films exhibited a typical viscoelastic behavior of amorphous polymers.

**Figure 5:**
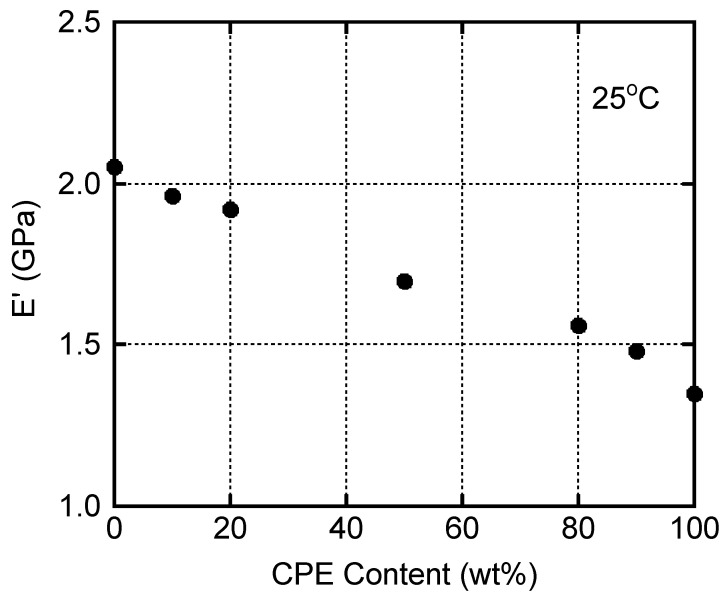
Relationship between *E*′ at 25 °C and CPE content.

As can be seen in the figure, *E*′ showed a slight decrease in the β relaxation region from −150 to 25 °C, ascribed to the local motion [39,40,41,42,43], which is sometimes called γ relaxation for PC. Correspondingly, *E*″ had a broad peak due to β relaxation. The peak temperature of pure CPE was around −60 °C, whereas that of PC was about −90 °C. It is interesting to note that the peak temperature of the PC-rich blend, i.e., PC/CPE (80/20), was almost the same as that of pure CPE, although the peak height was low. The peak area of *E*″ at the β relaxation, i.e., relaxation strength, increased with an increase in the CPE content. Because of the large relaxation strength, the modulus decrease around β relaxation became prominent with an increase in the CPE content. As a result, the *E*′ values beyond the β relaxation decreased with an increase in the CPE content, as illustrated in Figure 5. Obviously, the *E*′ values at room temperature (25 °C) decreased monotonically with the CPE content.

The glass-to-rubber transition, i.e., α relaxation, was clearly detected for all sample films with a single sharp peak of *E*″, irrespective of the blend ratio. It is well known that the peak width represents the distribution of the relaxation time and, thus, becomes broad once the concentration fluctuation occurs in the system. Therefore, this result demonstrates that PC and CPE were miscible in the experimental blend ratios. In the high temperature range of the rheological transition region, the slope of the *E*′ curves decreased again. For example, PC showed a different slope in *E*′ at around 165 °C, and CPE had the same trend at 130 °C. This is reasonable because the rubbery region should be detected after the transition region. The *E*′ values at the lowest temperature in the rubbery region seemed to slightly decrease with an increase in the CPE content, e.g., 10 MPa for pure PC and 5 MPa for pure CPE. This could be attributed to the low entanglement molecular weight *M_e_* of PC, which was reported to be 1330–1780 [44,45]. A low *M_e_* leads to a high rubbery plateau modulus *G_N_*^0^. Poly(ethylene terephthalate) is also known to have a low *M_e_* (1170–1450) [44,45]. Although CPE contains ethylene terephthalate unit, the diol components must give a high *M_e_* of CPE as compared with PC. In fact, Asai et al. revealed that a polyester comprising 1,4-cyclohexanedimethanol has a high *M_e_* [46].

**Figure 6:**
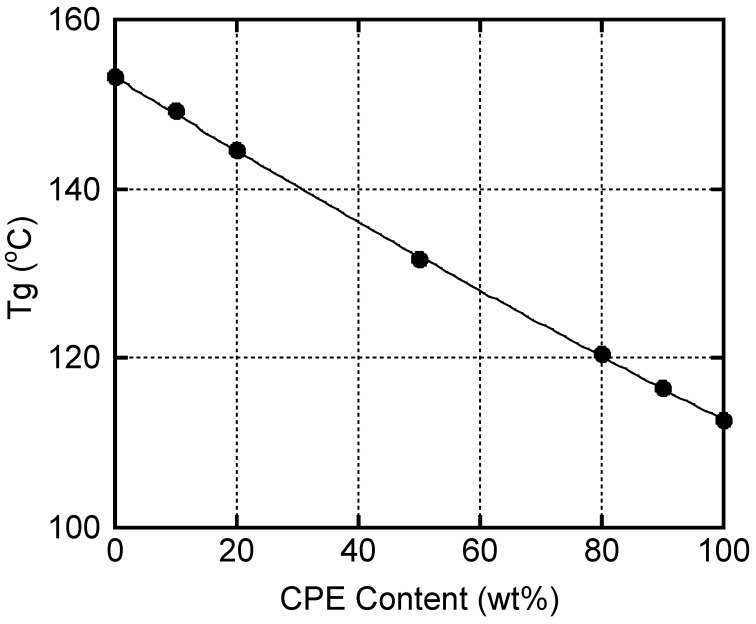
Relationship between *T_g_* and CPE content. The solid line represents the values calculated by the Fox equation.

The peak temperature of α relaxation was assigned as the glass transition temperature *T*_g_ in this study and plotted against the CPE content in Figure 6. The line in the figure represents the predicted values calculated from the Fox equation [47].

(3) $$\frac{1}{T_{g}\left(blend\right)}=\frac{w_{1}}{T_{g-1}}+\frac{w_{2}}{T_{g-2}}$$

where $w_{i}$ and *T_gi_* are the weight fraction and *T_g_* of the *i*-th component, respectively.

It is obvious that the experimental values of the blends corresponded well with those of the predicted ones. That is, *T_g_* of a blend can be controlled by the blend ratio.

### 3.4. Viscoelasticity in the Molten State

**Figure 7:**
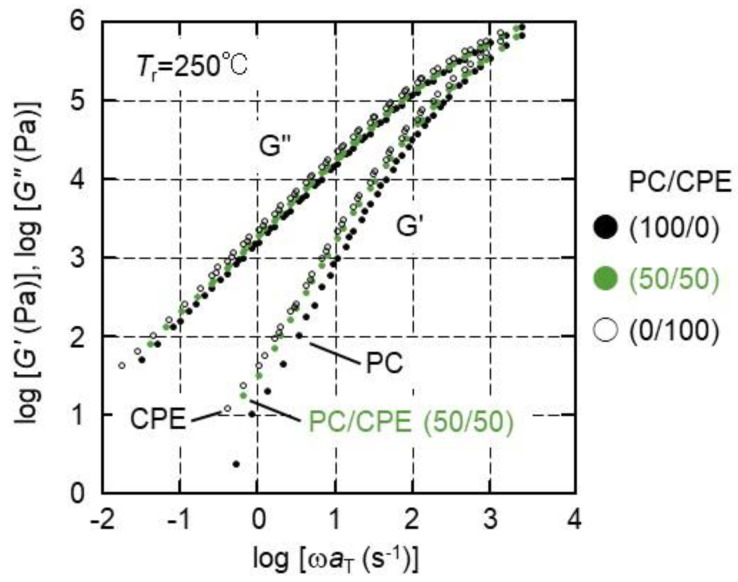
Master curves of angular frequency dependence of shear storage moduli *G*′ and loss moduli *G*″ for PC, CPE, and PC/CPE (50/50). The reference temperature was 250 °C.

Figure 7 shows the master curves of the angular frequency dependence of the shear storage moduli *G*′ and loss moduli *G*″. The reference temperature *T_r_* is 250 °C. It was found that both *G*′ and *G*″ of pure CPE were slightly higher than those of pure PC. The slopes of the *G*′ curves were approximately 2, not only for pure polymers, such as PC and CPE, but also for PC/CPE (50/50). It is well known that a contribution of interfacial tension appears in linear viscoelastic properties for a polymer blend with a phase-separated structure [48,49]. In particular, *G*′ in the low-frequency region is quite sensitive to prolonged relaxation ascribed to phase separation in general. In the present results, however, *G*′ of the blend did not show any shoulder. This result strongly supports that PC and CPE were miscible in the molten state.

Since the rheological terminal region was detected for both PC and CPE, the zero-shear viscosity *η*_0_, steady-state compliance *J_e_*^0^, and weight-average relaxation time *τ_W_* were calculated using the following relations:

(4) $$\eta_{0}=\underset{\omega \to 0}{\lim}\frac{G\prime \prime }{\omega }$$

(5) $$J_{e}^{0}=\underset{\omega \to 0}{\lim}\frac{G\prime }{G{\prime \prime }^{2}}$$

(6) $$\tau_{W}=\eta_{0}\;J_{e}^{0}$$

These values were summarized as follows; *η*_0_ = 1.6 × 10^3^ Pa s, *J_e_*^0^ = 4.2 × 10^−6^ Pa^−1^, and *τ_W_* = 6.5 × 10^−3^ s for PC; *η*_0_ = 2.2 × 10^3^ Pa s, *J_e_*^0^ = 8.1 × 10^−6^ Pa^−1^, and *τ_W_* = 1.8 × 10^−2^ s for CPE; and *η*_0_ = 1.8 × 10^3^ Pa s, *J_e_*^0^ = 8.9 × 10^−6^ Pa^−1^, and *τ_W_* = 1.6 × 10^−2^ s for PC/CPE (50/50). It was found that *η*_0_ of PC/CPE (50/50) was between those of the individual pure components, although *J_e_*^0^ of PC/CPE (50/50) was almost the same as that of pure CPE and higher than that of pure PC. A slightly high value of *J_e_*^0^ of the blend can be explained by the broad distribution of the relaxation time. As is well known, these rheological parameters can be described by the relaxation spectra *H*(*τ*) as follows:

(7) $$\eta_{0}=\overset{\infty }{\underset{-\infty }{\int }}H\left(\tau \right)\;\tau \;d\;\ln\tau$$

(8) $$J_{e}^{0}=\frac{\overset{\infty }{\underset{-\infty }{\int }}H\left(\tau \right)\;\tau^{2}\;d\;\ln\tau }{{\left(\overset{\infty }{\underset{-\infty }{\int }}H\left(\tau \right)\;\tau \;d\;\ln\tau \right)}^{2}}$$

Therefore, *J_e_*^0^ is determined by the relaxation time distribution. In particular, a long relaxation time mechanism greatly affects the value [50,51], and for a linear polymer, the molecular weight distribution decides it.

According to the classical theory [52], *J_e_*^0^ of a monodispersed polymer is inversely proportional to *G_N_*^0^. Assuming that PC and CPE have the same relaxation time distribution, *G_N_*^0^ of PC is almost twice as high as that of pure CPE. This prediction is not accurate when the relaxation time distribution, i.e., molecular weight distribution, has even a small level of experimental errors. However, it qualitatively agreed with the result in Figure 4, i.e., the difference in the *E*′ values at the lowest temperature in the rubbery region. Considering a significantly low *M_e_* value of PC, this result indicates that *M_e_* of CPE is also relatively low.

Furthermore, Figure 7 demonstrates that the time-superposition principle was applicable to PC/CPE (50/50), indicating that the phase separation did not take place in the measurement temperature range, i.e., from 230 to 280 °C. Furthermore, the flow activation energies were calculated by the Arrhenius plot of the horizontal shift factor *a_T_*. They were 111.4 kJ/mol for PC, 109.8 kJ/mol for CPE, and 110.6 kJ/mol for PC/CPE (50/50). The flow activation energy, i.e., the temperature sensitivity of the rheological properties, was not so different for all samples.

### 3.5. Mechanical Properties of the Films

**Figure 8:**
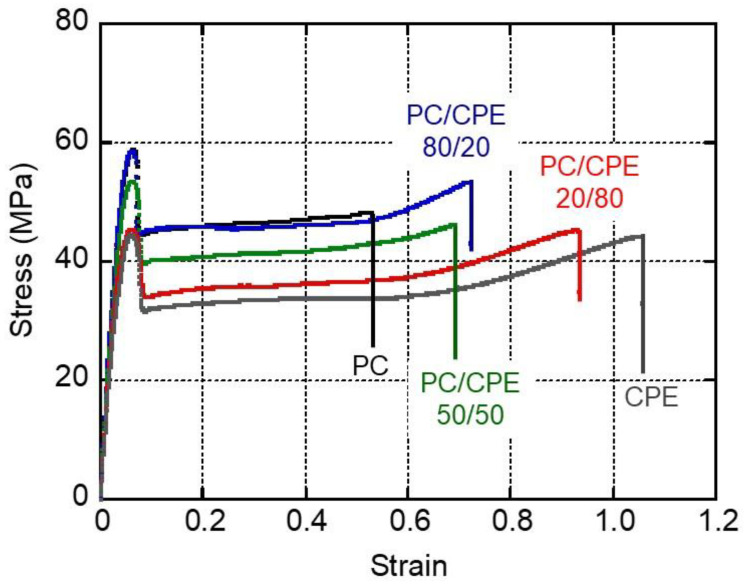
Stress–strain curves of the films.

Figure 8 shows the tensile stress–strain curves of the films, in which both stress and strain are nominal values. It was found that the PC and CPE films showed ductile behavior, demonstrating that they exhibited good mechanical toughness as previously reported [20,27]. It is well known that the mechanical toughness of PC is attributed to its molecular characteristics [53]. PC has a low *M_e_* and a low characteristic ratio. When *M_e_* is low, the polymer shows a high critical stress for crazing. Furthermore, a low critical stress for shear yielding is expected when the characteristic ratio is low. Therefore, PC tends to dominantly show shear yielding, leading to ductile behavior. The good mechanical toughness of CPE must also be attributed to a relatively low *M_e_*.

**Figure 9:**
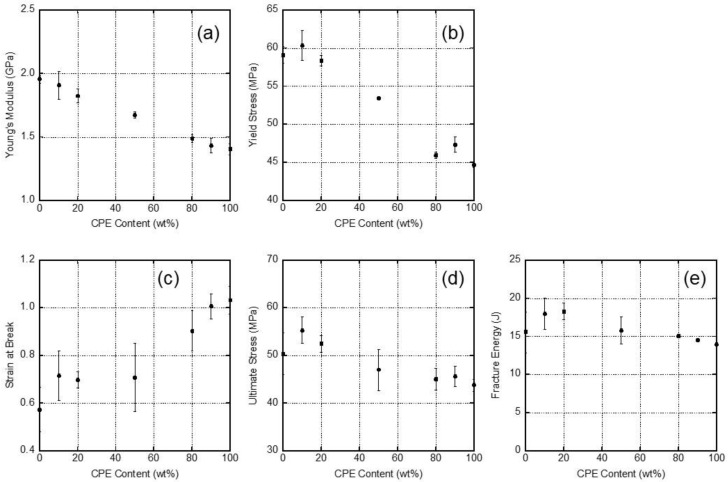
Tensile properties as a function of the CPE content: (**a**) Young’s modulus, (**b**) yield stress, (**c**) strain at break, (**d**) ultimate stress, and (**e**) fracture energy.

Young’s modulus, yield stress, strain at break, ultimate stress, and fracture energy, (i.e., the area of a stress–strain curve), are summarized in Figure 9 with experimental error bars. The initial modulus decreased with the CPE content, which corresponded with the tensile storage modulus *E*′ at 25 °C, shown in Figure 5. The yield stress also decreased with the CPE content. In contrast, the strain at break increased with the CPE content. Regarding the ultimate stress and fracture energy, there was no specific trend. This is plausible because these values were almost similar for PC and CPE.

It can be concluded that the mechanical properties of the blend films are intermediate between those of individual pure polymer films. Moreover, it should be noted that all blend films exhibit good mechanical toughness with excellent transparency. Considering that PC shows poor flowability at injection molding, the blends can be applicable to a large injection-molded product because of the viscosity drop in a mold [15,54,55]. When the shear viscosity greatly decreases, it becomes possible for PC to add fillers in order to enhance the modulus and surface hardness [56]. This will expand applications. Moreover, PC has other problems [1,2,3], such as high birefringence, anti-solvent resistance, and scratch resistance. These properties should be examined for the blends in the near future to identify appropriate applications.

## 4. Conclusions

A new type of transparent copolyester, CPE, which is commercially available, was found to be miscible with PC irrespective of its blend ratios in both the solid and molten states. Because the blend films did not show phase separation, they were transparent. Moreover, their *T_g_*’s can be predicted by the Fox equation. The mechanical properties of the blend films were also determined by the blend ratio; i.e., Young’s modulus and yield stress decreased with the CPE content, while the strain at break increased with the CPE content. It was suggested that *M_e_* of CPE is relatively low, leading to good mechanical toughness. As a result, all blend films, including pure CPE, exhibited good mechanical toughness.

These results demonstrate that a new type of transparent glassy polymer film with high mechanical toughness can be prepared by the melt blending of PC and CPE with no chemical reaction. Because the melt viscosity and *T*_g_ can be easily controlled, various processing operations are available for the blends. Moreover, the thermal and mechanical properties of the films are only adjustable by the blend ratio. This must be a great benefit for various applications.

## Data Availability

Not available.
